# Measles Outbreak in a Highly Vaccinated Population — Israel, July–August 2017

**DOI:** 10.15585/mmwr.mm6742a4

**Published:** 2018-10-26

**Authors:** Eva Avramovich, Viki Indenbaum, Meital Haber, Ziva Amitai, Evgeny Tsifanski, Sahar Farjun, Alona Sarig, Adi Bracha, Karina Castillo, Michal Perry Markovich, Inbal Galor

**Affiliations:** ^1^Israeli Defense Force Medical Corps; ^2^Central Virology Laboratory, Public Health Services, Israel Ministry of Health; ^3^Tel Aviv Health District, Israel Ministry of Health; ^4^Veterinary Services, Israel Ministry of Agriculture and Rural Development.

On August 6, 2017, the Israeli Defense Force Public Heath Branch (IDFPHB) was notified of two suspected measles cases. IDFPHB conducted an epidemiologic investigation, which identified nine measles cases in a population with high measles vaccination coverage. All measles patients had signs and symptoms consistent with modified measles (i.e., less severe disease with milder rash, fever, or both, with or without other mild typical measles symptoms). A total of 1,392 contacts were identified, and 162 received postexposure prophylaxis (PEP) with measles-mumps-rubella (MMR) vaccine; the remaining contacts were followed for 21 days (one incubation period). No tertiary cases were identified.

## Investigation and Findings

The first two cases (in patients A and B) were reported on August 6, 2017. Both cases occurred in soldiers who developed mild symptoms (fever and maculopapular rash) on August 4 and subsequently were hospitalized in isolation at a civilian hospital. Urine and serum specimens were sent to Israel’s National Measles and Rubella Laboratory (NMRL). Neither patient had traveled or had a known exposure to measles, nor was any epidemiologic link evident between the cases. Both patients had self-reported history of receipt of 2 doses of MMR vaccine. The cases in patients A and B were laboratory-confirmed serologically and by urine polymerase chain reaction (PCR) testing on August 7 and August 9, respectively (Table). A third patient with mild symptoms (patient C) was reported by NMRL staff members to IDFPHB on August 7 after urine PCR confirmation. Patient C, a soldier aged 19 years, was the partner of patient A and reported having received 2 doses of measles vaccine.

**TABLE:** Age, measles vaccination history, and laboratory results for nine patients with measles — Israel, July–August, 2017

Patient	Age (yrs)	Measles vaccination history	Rash onset date	Urine PCR	IgM	IgG	Avidity^†^	Confirmation
Primary	21	3 doses*	Jul 24	Not available	Positive	Positive	62	Confirmed
A^§^	20	2 doses^¶^	Aug 4	Positive	Positive	Negative	Not applicable	Confirmed
B	20	2 doses^¶^	Aug 5	Positive	Positive	Positive	70	Confirmed
C	20	2 doses^¶^	Aug 6	Positive	Negative	Positive	77	Confirmed
D^§^	20	2 doses^¶^	Aug 4	Negative**	Equivocal	Positive	74	Confirmed
E	37	2 doses^††^	Aug 7	Negative**	Negative	Positive	71	Probable
F	19	2 doses*	Aug 7	Positive	Equivocal	Positive	65	Confirmed
G	19	2 doses*	Aug 7	Positive	Positive	Positive	75	Confirmed
H	19	2 doses*	Aug 10	Positive	Negative	Positive	76	Confirmed

**Abbreviations:** IgG = immunoglobulin G; IgM = immunoglobulin M; PCR = polymerase chain reaction.

* Documentation of vaccination.

^†^ >60% = high avidity.

^§^ Ring vaccination (vaccination of all susceptible persons in the unit) was performed.

^¶^ Reported history of vaccination.

** PCR sample not appropriately transported to laboratory.

^††^ 1 dose documented; second reported.

IDFPHB undertook an epidemiologic investigation to determine the source of infection, to identify contacts, and to recommend PEP. Because all three cases appeared within days of one another, a common source of infection was suspected.

Investigators learned that patients A, B, and C had visited the same crowded, mixed civilian-military clinic on July 24 during the same hour; therefore, the clinic was suspected to be the site of exposure. To evaluate that hypothesis, investigators reviewed medical records of all patients treated at the clinic on July 24 during the same time that patients A, B, and C visited. One patient examined in the clinic was a Ukraine-born soldier, aged 21 years, who was evaluated for fever and rash; measles was not suspected at the time. The investigation found that he had returned to Israel 3 days before visiting the clinic, having traveled to three European countries (France, Germany, and Ukraine) with ongoing measles outbreaks (*1*). Because he was suspected to be the primary patient, his serum specimen was forwarded to NMRL, where measles was serologically confirmed.

The primary patient would have been infectious from approximately 4 days before until 4 days after the onset of rash. Because more than 14 days had passed from the July 24 clinic exposure, it was anticipated that some exposed clinic contacts (in addition to patients A, B, and C) might have already developed measles. Therefore, the medical records of all soldiers who went to the clinic on July 24, as well as other military contacts of the primary patient, were reviewed daily for the 21 days beginning July 24. The aim was to identify occurrence of fever, rash, conjunctivitis, or Koplik spots among the contacts.

For the purpose of the investigation, a suspected case of measles was defined as the presence of a febrile rash illness. Confirmed measles was defined as positive test for measles by urine PCR or positive/equivocal measles immunoglobulin M, and a probable case was defined as a suspected case with suggestive laboratory results (positive immunoglobulin G [IgG] with high IgG avidity, indicative of a past immunologic response to measles vaccine or infection).

The review of medical records led to further evaluation of 14 patients, including vaccination history, exposure history, inquiries regarding potential contacts, and a physical examination by a general practitioner. Among the 14 patients, eight measles cases were identified in addition to the primary case, seven of which were laboratory-confirmed (Table). The median patient age was 20 years (range = 19–37 years). All patients had mild disease with rash, fever, or both and minimal or no conjunctivitis or Koplik spots, consistent with modified measles (*2*). There were no known complications. Two patients (A and B) were hospitalized, primarily to establish the diagnosis and provide isolation.

Patients were residents of central Israel and served on different military bases. Four patients had documentation of receipt of 2 doses of measles-containing vaccine, four reported receiving 2 doses during childhood (with no documentation), and the primary patient had documentation of receipt of 3 doses of MMR in Ukraine, one each in 1997, 1998, and 2002. Patient A’s report of receipt of 2 doses of measles-containing vaccine was inconsistent with the negative IgG test result. Laboratory testing confirmed high avidity (>60% IgG) in all patients except patient A (Table), suggesting a previous immune response (*3*). The epidemiologic investigation identified 1,392 contacts of these nine patients. No measles cases were diagnosed among contacts of patients A–H.

Phylogenetic analysis of virus isolates from three patients identified B3 genotype. Concurrent with this outbreak, three measles cases with no apparent connection to the military cases were diagnosed in civilians in the Tel Aviv district. Measles genotype B3 was identified in these cases as well, and the sequence of 450nt region of the N gene was identical to that from the military isolates, supporting the suspicion of epidemiologic linkage among all Israeli cases. According to GenBank, the U.S. National Institute of Health’s open-access, annotated collection of all publicly available nucleotide sequences, measles virus B3 genotype with the identical 450nt N gene sequence was detected in Hungary (MG323532.1), Germany (MF593154.1), Belgium (MF490426.1), and Italy (KY801730.1) during the same period, strengthening the evidence that the primary case was acquired while traveling in Europe.

## Public Health Response

During the active search for contacts of patients A–H, a total of 1,392 military contacts were identified and located, and their physicians were notified. All contacts were instructed to seek medical care or call IDFPHB if they developed fever or rash during the subsequent 21 days. Contacts identified within 72 hours of exposure who had received fewer than 2 doses of MMR vaccine were given an MMR dose for PEP. By August 27, a total of 162 soldiers had received PEP with MMR vaccine. Among the remaining contacts who were not offered PEP, most had documentation of receipt of 2 doses of measles-containing vaccine; some were not identified until >72 hours after exposure. Because of the crowded nature of the military units of two patients (A and D), vaccination of all susceptible persons in the unit (ring vaccination) was performed. After consultation with Israel’s Ministry of Health (MOH) public health and laboratory specialists, and because of the low attack rate, a decision was made to not recommend a third MMR dose for contacts. No quarantine was recommended for contacts.

As mandated, IDFPHB notified MOH about the cases. Because more than 2 weeks had elapsed from the airline flight taken by the primary patient until the diagnosis of measles was made, it was not possible to administer PEP to the passengers of the flight. MOH was not notified of any Israeli measles cases among contacts from the flight. Additional measures taken during the outbreak were confirmation of vaccination status of health care workers in the clinics and in military units with confirmed cases and issuance of an alert for military health care practitioners about the outbreak.

## Discussion

This measles outbreak occurred in an adult population with high 2-dose measles vaccination coverage. The primary patient had documentation of receipt of 3 doses of measles-containing vaccine, one each at ages 1, 2, and 6 years, per the vaccination schedule in Ukraine. Although it is possible that the vaccination record contained an error, the high IgG avidity suggests secondary vaccine failure (*2*). All patients except one had high measles IgG avidity, which is an indicator of previous vaccination or previous infection. Because all the serum specimens (except that from the primary patient) were collected 2–3 days after the onset of symptoms, the high avidity IgG was assumed to be a result of patients’ previous vaccination.

Although outbreaks of measles among vaccinated populations have been reported worldwide (*4*–*7*), most outbreaks in Israel have occurred in unvaccinated or partially vaccinated populations (*8*,*9*). Measles transmission from a vaccinated person with documented secondary vaccine failure also has been described in New York City in 2011, including among vaccinated health care providers (*4*), and in the Marshall Islands (*10*). Waning of vaccine-induced immunity is a phenomenon that needs to be addressed, especially in regions where circulation of wild measles virus is low. Further studies, which might include seroepidemiologic studies of the dynamics of IgG levels by age, are needed to assess measles immunity and incidence of measles in populations with high 2-dose vaccination coverage. Demonstrating waning immunity with age could guide development of recommended vaccination regimens.

This outbreak highlights the importance of a thorough epidemiologic and laboratory investigation of suspected cases of measles, regardless of vaccination status, as well as the need for active surveillance of contacts. The symptoms reported by patients with secondary measles cases were modified from the typical signs of fever; rash; and coryza, conjunctivitis, or cough. Without active surveillance, the possibility of measles likely would not have been considered, and circulation of the virus might have continued. Health care providers should include measles in the differential diagnosis of fever and rash even in a vaccinated patient and obtain appropriate laboratory testing.

Absence of tertiary cases in this outbreak is consistent with the lower risk for transmission reported in other cases of measles in vaccinated persons, possibly owing to their milder symptoms, including lack of or reduced cough (*4*,*5*). In this outbreak, most contacts being fully vaccinated probably contributed to rapid containment.

**Summary**

**What is already known about this topic?**

Measles occurs sporadically in Israel and can be imported by travelers. Measles outbreaks occurred in 15 European countries during the summer of 2017.

**What is added by this report?**

During July and August 2017, nine measles cases occurred among vaccinated Israeli soldiers. The primary patient had recently traveled to Europe. All other cases occurred in his direct contacts. All patients had mild illness; no tertiary cases occurred.

**What are the implications for public health practice?**

Modified measles might not be suspected in persons with documentation of vaccination. In outbreak settings, health care providers should maintain a high index of suspicion for measles, even in vaccinated patients, and conduct a thorough epidemiologic and laboratory investigation of suspected measles cases.
